# Sea Voyages for Inebriety

**Published:** 1901-11-02

**Authors:** 


### SEA VOYAGES FOR INEBRIETY.

At the last meeting of the Society for the Study of Inebriety, Dr. Wynn Westcott read a paper in which he gave expression to certain views in regard to the use of sea voyages in the treatment of inebriety which should be carefully considered by all physicians who may feel inclined, as no doubt many do at times feel inclined, to advise a reformed drinker to take a sea voyage as a means of establishing his health before returning again to his ordinary mode of life. Dr. Wynn Westcott said that he had formed a decided opinion on the subject. The sort of cases which would be likely to be sent would be those in which it was desirable to re-establish the general health, and it was to be remembered that under the most favourable circumstances life at sea was not all rest and comfort, and that the nursing, dieting and quietude which were essential for patients with perhaps fatty heart or liver, were very seldom to be obtained. Few cases, indeed, of advanced disease of any kind seemed to him suitable for sea-life, even at its best. There was no doubt, moreover, that the dulness and monotony of the life, and other causes, tended to produce thirstiness; often a good deal was drunk just before reaching the home port in consequence of the people on board being in a disturbed, excited state; and again the periods of nervous irritability, depression, and præcordial distress to which the dipsomaniac was subject, were likely to be more frequent on board ship than ashore. Therefore, if a dipsomaniac really wanted to get well, he should be discouraged from taking a sea voyage. The ideal ship for a patient who wished to travel in this manner would be a sailing ship which was commissioned on teetotal principles, with a teetotal captain and crew, and which touched at scarcely a single port *en route*. Such a ship, however, would, we think, be generally difficult to find. In any case no reformed drunkard should be trusted on a sea voyage *alone*.

